# Mapping the Hidden Landscape of Ln-Rich Platinides

**DOI:** 10.1021/acs.inorgchem.6c03130

**Published:** 2026-08-24

**Authors:** Volodymyr Smetana, Vitalii Shtender, Gerd H. Meyer, Anja-Verena Mudring

**Affiliations:** † Department of Biological and Chemical Engineering and iNANO, 1006 Aarhus University, Aarhus C, 8000 Aarhus, Denmark; ‡ Department of Chemistry—Ångström Laboratory, 8097 Uppsala University, P.O. Box 538, SE-751 21 Uppsala, Sweden; § Department of Physics, 8075 Umeå University, 90187 Umeå, Sweden

## Abstract

Despite extensive explorations, lanthanide-platinum (Ln-Pt) systems showed surprising differences with respect to reported compounds, particularly in the Ln-rich area. A re-examination of the corresponding systems revealed several hidden series with light Ln up to Gd, namely Ln_2_Pt (Pearson symbol *oP*12, Co_2_Si structure type, space group *Pnma*), Ln_5_Pt_3_ (Pearson symbol *tP*32, Pu_5_Rh_3_ structure type, space group *P*4/*ncc* or Pearson symbol *hP*16, Mn_5_Si_3_ structure type, space group *P*6_3_/*mcm*), Ln_3_Pt_2_ (Pearson symbol *hR*45, Er_3_Ni_2_ structure type, space group *R*3̅) and Ln_3_Pt_2–*x*_ (Pearson symbol *tI*80–16*x*, Pr_3_Pt_2–*x*_ structure type, space group *I*4/*mcm*). All crystal structure and phase analyses have been performed by means of single-crystal and powder X-ray diffraction. A comprehensive comparative analysis of the related binary systems in the range of 33–40 at. % of the transition metal has been performed. Notably, none of the series is identical to any other in this work or other Ln-transition metal systems. DFT-based electronic structure calculations were employed to examine the structural preferences of the selected series and to investigate possible polymorphic transformations.

## Graphical abstract

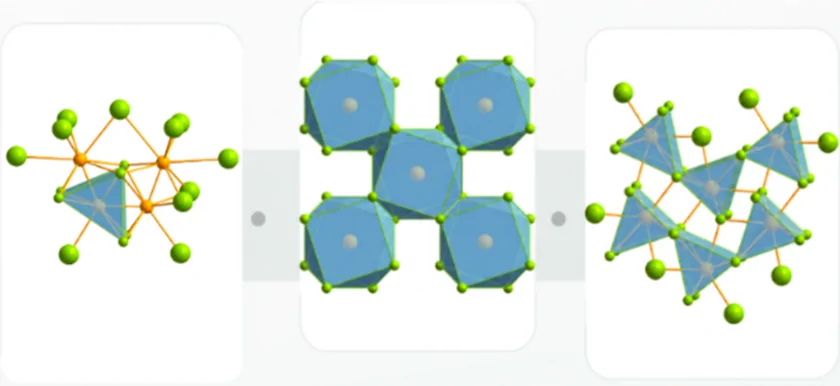

## Introduction

The position of platinum in the sixth period of the periodic table endows this element with electronic and chemical properties that noticeably distinguish it from its lighter group 10 counterparts similar to gold.^1^ These differences arise primarily from pronounced relativistic effects influencing the electronic structure of platinum, particularly its valence states.^2,3^ The relativistic contraction of the 6s orbital and the concomitant destabilization of the 5d orbitals lead to an enhanced inertness and high cohesive energy, making Pt both chemically noble and physically robust. This stabilization also contributes to platinum’s relatively high electronegativity and strong preference for low oxidation states compared to lighter homologues such as palladium or nickel.^3^ Consequently, platinum readily forms a range of intermetallic compounds and complexes that feature significant metal–metal bonding or partial charge transfer involving Pt atoms.^4–6^ A ground-breaking example is the formation of salt-like platinum compounds with alkali metals, particularly Cs, featuring Pt^2–^ anions^7,8^ in analogy with metal chalcogenides.^9^

Although platinum intermetallic compounds often exhibit electronic analogies with those of gold, reflecting the shared influence of relativistic effects on bonding and crystal structure formation, their diversity of reported structures is less explored.^10^ On the other hand, the ability of Pt to adopt diverse coordination environments and to participate in extended Pt–Pt networks enables a rich and tunable structural chemistry, offering opportunities for the design of materials with novel electronic properties that remain far from fully understood. The most prominent examples of relativistic influence include topological features such as Dirac node arcs in PtSn_4_,^11^ topological semimetality in Pt_3_Sn or Pt_3_Sn_1–*x*_Fe_*x*_ ^12^ or surface superconductivity in LnPtBi or PtBi_2_.^13,14^ When combined with magnetic elements, unconventional magnetic behavior is also frequently observed from field-induced Weyl magnetism and noncollinear spin textures to the anomalous Hall effect.^15–19^

In this light, binary lanthanide-platinum (Ln-Pt) systems are particularly interesting as they provide a platform for exploring the interaction between localized 4f magnetism and relativistic d-electron states.^20^ In these systems, the large magnetic moments of the lanthanides are coupled predominantly through Pt-derived conduction electrons, enabling indirect exchange interactions of the RKKY type that are highly sensitive to the strong spin–orbit coupling inherent to platinum.^21^ The absence of additional chemical components in binary Ln-Pt compounds minimizes structural and electronic complexity, highlighting the intrinsic roles of 4f–5d hybridization and relativistic effects.

The binary lanthanide-transition metal systems were intensively investigated in the mid-20th century, and the corresponding phase diagrams were reported.^10^ However, as we discovered recently, the present information was frequently incomplete^22–26^ due to limitations of X-ray analysis of that time, hampered by the nobility of Pt, high-melting temperatures of the given compounds, combined with enormous compositional diversity and sometimes the presence of low-temperature polymorphs not accessible using conventional methods. For instance, Pr_4_Pt_3_ could not later be reproduced using prolonged annealing without Pr halide fluxes.^22^ In this work, we report four series of overlooked Ln-rich platinides, which include five different structure types and cover the gray zone of the corresponding Ln–Pt binary systems.

## Experimental Section

### Synthesis

All samples were prepared using pure metals in the form of Ln (at least 99.99 wt %, HEFA Rare Earth Canada Ltd.) and Pt pieces (99.999 wt %, Neyco). Loadings corresponding to Ln_3_Pt_2_, Ln_2_Pt and Ln_5_Pt_3_ and a total mass of 300–400 mg each were arc melted in Ar atmosphere with Zr ingot as a getter. All samples were remelted multiple times, turning the ingots upside-down to ensure better homogenization. No weight losses exceeding 1% have been observed in any of the samples from La to Nd. The reactions with Sm and Eu were particularly challenging, as these elements’ boiling points lie significantly below the melting point of Pt, leading to notable mass losses and nominal composition shifts. The as-cast samples were examined with powder X-ray diffraction (PXRD). Afterward, samples were placed in Ta foil, sealed in fused silica, and annealed at 800–1000 °C for 10 days. Samples with Eu were annealed at 700 °C. Subsequently, the ampules were quenched in cold water. Single crystals of sufficient quality could be isolated from all crushed samples, although not for all compounds. Additional Eu–Pt samples were placed in sealed Ta crucibles, which were in turn sealed in stainless steel tubes under Ar. The samples were prereacted at 1000 °C for 2 h and subsequently cooled to room temperature in cold water.

### X-ray Diffraction

The purities of the products were established through powder diffraction (PXRD). Powder patterns were collected using the STOE STADI P powder diffractometer (transmission geometry, Ag Kα1 radiation) in the 2–40° 2Θ range, with 0.02° 2Θ step and 30 s per step. Phase analyses were performed with the WinXPow 3.2 program (STOE and Cie GmbH). The TOPAS 6 software package was used for Rietveld analysis of the collected data sets.^27^

Single-crystal X-ray diffraction (SCXRD) analyses were performed at room temperature (295(2) K) using STOE STADIVARI diffractometer operating at 60 kV 1.4 mA equipped with a Dectris Pilatus detector and an Incoatec IμS Mo microfocus source. The raw data were collected and handled with the STOE X-Area software package (STOE and Cie GmbH). Initial models of the crystal structures were derived using SHELXT-2018 and refined with SHELXL-2018 within the APEX4 software package. Anisotropic displacement parameters have been refined for all atoms. Reflection intensities and symmetries were carefully re-examined with the aid of the PLATON program to exclude any missing higher symmetry.^28–31^ Details of the structural refinements have been collected in Tables S1 and S2. The program DIAMOND was used for drawing and analyzing the crystal structure.^32^

### Energy-Dispersive X-ray Spectroscopy

For compositional inspection and complementary qualitative phase analysis, energy-dispersive X-ray (EDX) spectroscopy was used. The analysis has been performed using preselected single crystals of Nd_3_Pt_2–*x*_. Compositional analysis was performed with a JEOL 7000F scanning electron microscopy system equipped with an Oxford Inca X-sight energy dispersive spectrometer at an acceleration voltage of 20 kV. The samples were mounted on a Bakelite© support. Microscopic examinations were carried out in the backscattered electron mode. For elemental analysis, the Nd L-alpha and Pt L-alpha edges were used. Minor Pt deficiency, as well as the absence of any major stabilizing impurities, have been detected within the accuracy of the method (Figure S1).

### Computations Details

First-principles calculations were carried out using the density functional theory (DFT) implemented in the Vienna ab initio simulation program (VASP) package (Version 6.4.2)^33–37^ with the generalized gradient approximation (GGA) exchange–correlation functional,^38^ under the Projector Augmented Wave (PAW) functions.^39^ The calculations included 16 valence electrons for Pt, while the Ln_3 potentials were used for the lanthanide atoms, treating 4f electrons as core states. A plane-wave-expansion cutoff energy of 550 eV was used for all the calculations. Preliminary structural relaxation was carried out until the residual atomic forces were less than 0.02 eV Å^–1^. Structural relaxation calculations were done with a 6 × 6 × 8 k-mesh and an energy convergence criterion of 10^–7^ eV. Static Total-energy calculations were subsequently performed on the relaxed structures to compare the relative stability of the hexagonal and tetragonal forms of Ln_5_Pt_3_. Optimized unit cell parameters have been summarized in Table S3. Phonon calculations were not performed; therefore, the calculated energies describe the relative static stability of the considered structure types and do not constitute an assessment of their dynamic stability. The crystal orbital Hamilton population (COHP) method has been applied to analyze the bonding picture using VASP calculations and LOBSTER program for data extraction.^40,41^

## Results and Discussion

New compounds belonging to three isocompositional series and five structure types have been identified and structurally characterized for the light lanthanides up to Gd: Ln_3_Pt_2_ (Er_3_Ni_2_ type), Ln_3_Pt_2–*x*_ (Pr_3_Pt_2–*x*_ type), Ln_5_Pt_3_ (Pu_5_Rh_3_ and Mn_5_Si_3_ types) and Ln_2_Pt (Co_2_Si type) compounds (see Tables 1 and S4 in the Supporting Information for comparison of unit cell data).

**Table 1:**
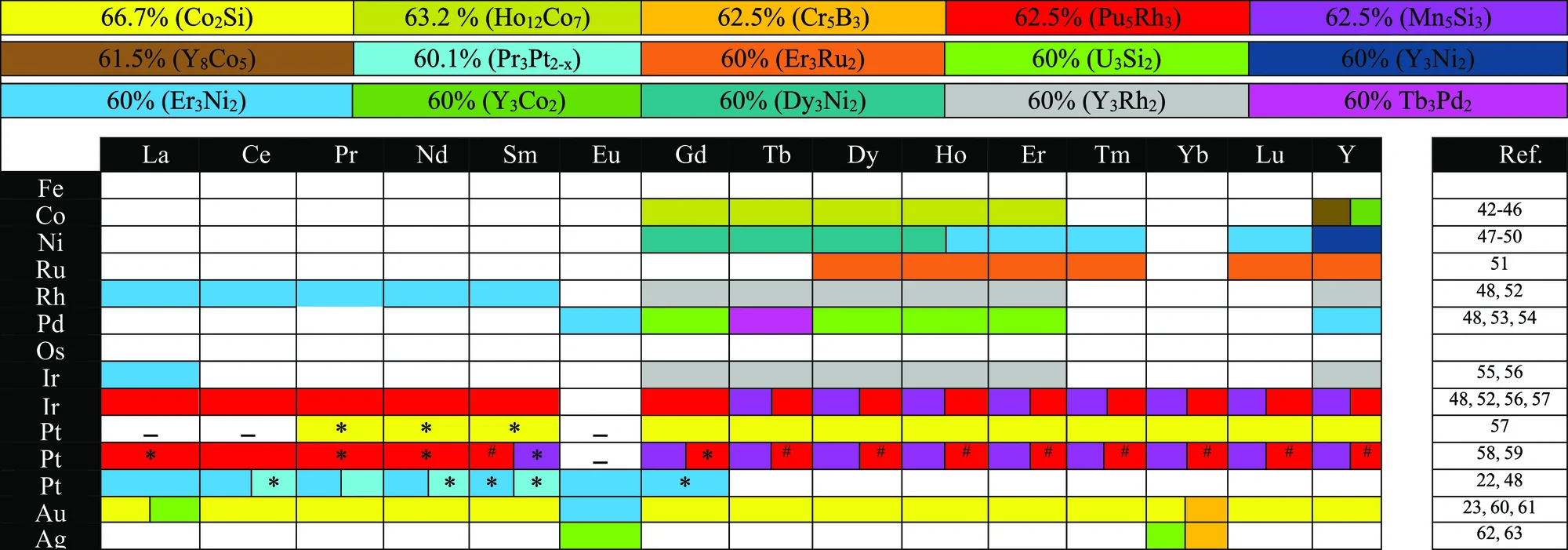
Overview of the Compounds and Their Structures Observed Around 60–70 at. % *R* in the *R*–*T* Binary Systems^42–45,48,49,51,53,55–58,60,61,63,77,89,90^ ^,^ ^a^

a *—experimentally found in this work; #**—**DFT-based, see Table 2.

A Pt-deficient binary phase Pr_3_Pt_2–*x*_ was previously reported during the search for low-temperature lanthanide-transition-metal binary intermetallics using NaCl as flux. This approach has proven highly effective for revealing thermodynamically stable but kinetically obscured low-temperature polymorphs.^24^ Later, Pr_3_Pt_2–*x*_ was also observed in arc-melted samples annealed at 1050 °C, together with the reported stoichiometric Pr_3_Pt_2_ phase.^22^ However, the phase was not obtained in high yield at that time, suggesting either a narrow stability range or possible phase-equilibrium limitations.

In the present study, the freshly prepared as-cast sample with nominal composition Pr_3_Pt_2_ appeared to contain a major fraction of Pr_3_Pt_2–*x*_ (Figure S1), thereby revealing the true stability area of Pr_3_Pt_2–*x*_ as a high-temperature (*ht*) polymorph. Considering the significant discrepancies among available binary phase diagrams^64^ and the frequent polymorphism observed in other Ln-T systems,^24^ we therefore took a closer look at the composition range near 60 at. % Ln in this and neighboring Ln-Pt systems up to Gd. As a result, 12 new compounds were experimentally confirmed within the composition range of 60–67 at. % Ln. Several additional phases were suggested to exist and were scrutinized by DFT calculations.

### Ln_3_T_2_ Family

The crystal structure of **Ln**_**3**_ **Pt**_**2–*x***_ can formally be assigned to the Gd_3_Ga_2_ structure type^65^ representing its first example without any main-group elements. This series has been observed for Ce, Pr, Nd and Sm but not for La, apparently owing to its larger atomic size. The structure can be described as an intergrowth of two alternating slabs (Figure 1). Slab A is planar and corresponds to the Ti_3_Co_5_B_2_ structure type,^66^ as reflected by its semiregular tiling of triangular, square, and pentagonal prisms centered by Pt (Pt@Ln_6_) and (Pt@Ln_8_) and Ln (Ln@Ln_10_) atoms, respectively. Slab B is more corrugated and consists of regular Pt@Ln_8_ square antiprisms and Pt_2_@Ln_10_ bicentered pentagonal antiprisms with displaced bases. However, the Ln atoms centering the Ln pentagonal prism in slab A also belong to the coordination sphere of these Pt positions. Thus, these polyhedra may alternatively be described as bicentered equatorially tetracapped tetragonal prisms with corrugated bases, or as fused tricapped trigonal prisms where Pt atoms simultaneously serve as capping atoms for one another (Figure 2).

**Figure 1:**
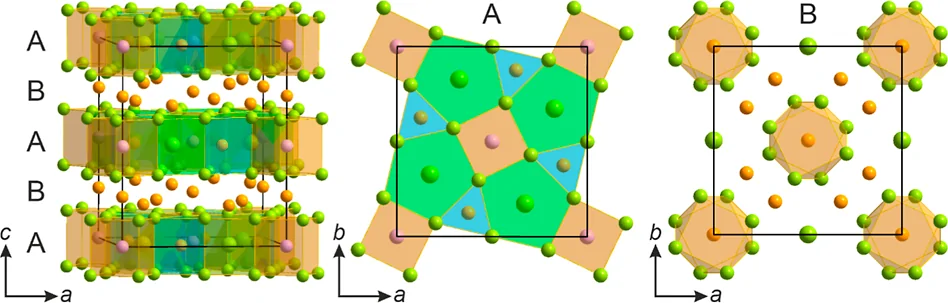
Atomic packing in the crystal structure of LnPt_2–*x*_ (left) and projections of the layers down the *c* axis. Y atoms are shown green, Pt—orange, and deficient Pt—lavender.

**Figure 2:**
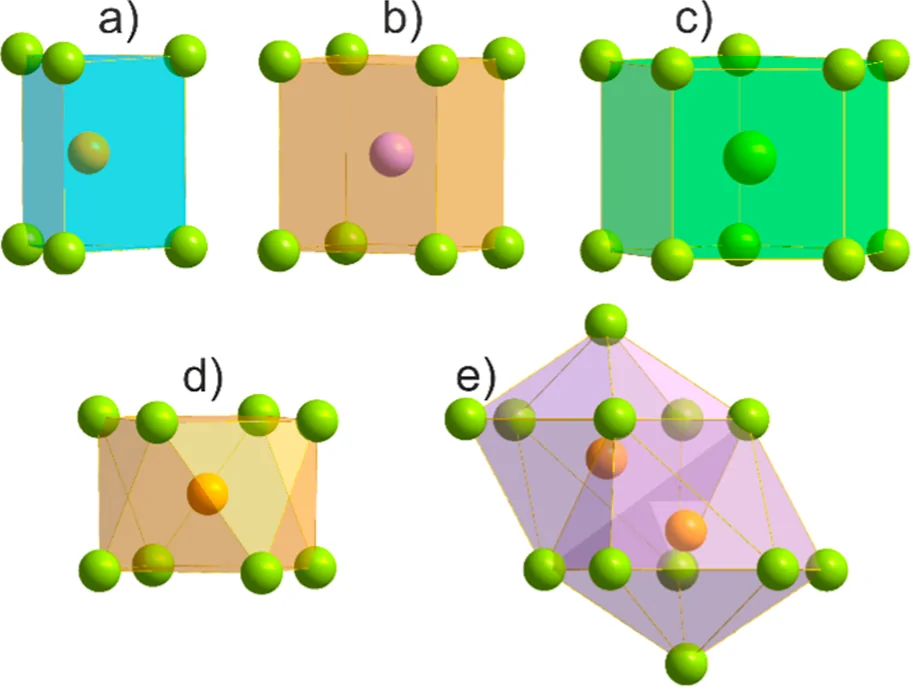
Polyhedral environment observed in the crystal structure of LnPt_2*x*_. (a) Pt@Ln_6_, (b) Pt@Ln_8_, (c) Ln@Ln_10_, (d) Pt@Ln_8_ and (e) Pt_2_@Ln_12_. Ln atoms are shown green, Pt—orange, and deficient Pt—lavender.

Although uniform pentagonal tilings are known, such as the Cairo tiling,^67^ ideal tilings involving pentagons, for example the 3.5.4.5 tiling, are generally not realized in crystalline solids because of symmetry restrictions. The Ti_3_Co_5_B_2_ structure type^66^ represents one of the closest approximants with slightly distorted triangles and pentagons. This motif is common in intermetallic borides, although it is often incorporated into larger monolayered patterns based on triangular, squares and pentagonal prisms.^68,69^ By contrast, the Gd_3_Ga_2_ structure type^65^ belongs to a different family of structures, in which layers with Archimedean-like tiling alternate with unrelated intermediate slabs. Notably, all previously reported compounds of this type share a characteristic feature: the presence of at least one group 13 element, excluding boron.^10^

**Ln**_**3**_ **Pt**_**2**_ crystallize in the Er_3_Ni_2_ structure type,^70^ which is also adopted by several other compounds with transition metals. Therefore, only the basic structural details are described here, with emphasis placed on comparison within the Ln_3_T_2_ family. The crystal structure can be conveniently described using two construction motifs: linear Ln_*n*_ chains and (LnPt)_*n*_ helices in a 1:2 ratio (Figure 3).

**Figure 3:**
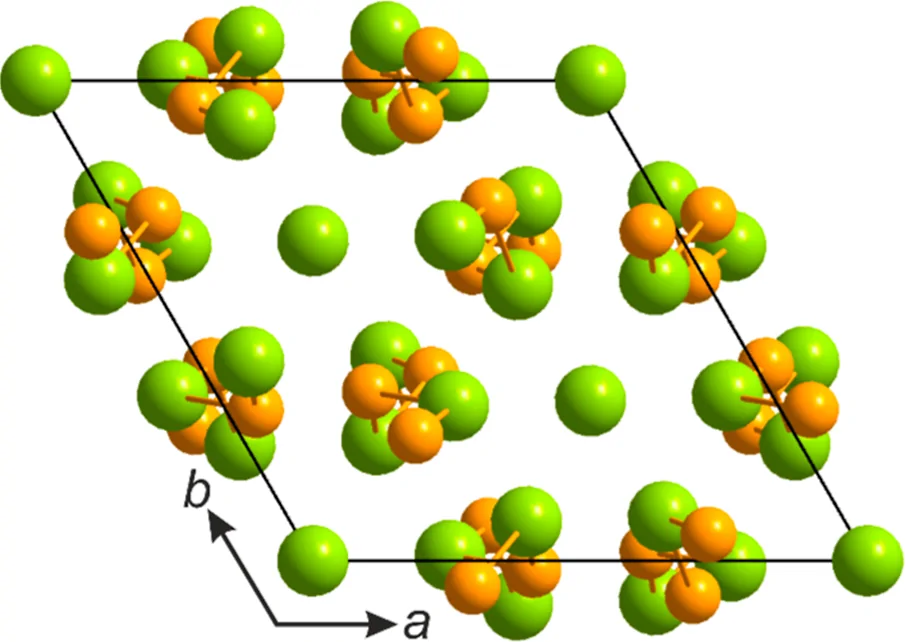
Projection of the crystal structure of Sm_3_Pt_2_ down the *c* axis. Sm atoms are green and Pt—orange.

Despite only minor compositional differences from Ln_3_Pt_2–*x*_, the structural changes are substantial and are clearly reflected in the more complex local atomic environments. Notably, no simple symmetry relationship could be identified between the two polymorphs. The Pt atoms adopt tricapped trigonal-prismatic coordination, whereas the Sm environments are considerably more diverse. Sm1 and Sm2 both have a coordination number of 16 and form strongly distorted Frank–Kasper polyhedra, while Sm3 is surrounded by a Sm_8_ cube with all faces capped by Pt atoms, corresponding to a tetrakishexahedron.

As in Ln_3_Pt_2–*x*_, Pt–Pt pairs with distances slightly below 3 Å are observed in Ln_3_Pt_2_, representing the most pronounced similarity between the two structures. These pairs lie at the cores of similar, although slightly more regular, bicentered equatorially tetracapped tetragonal prisms identical to those in Ln_3_Pt_2–*x*_ (Figure 2e). These prisms occupy a significant fraction of the structure and link Ln-Pt helices and Ln chains. However, their arrangement is less ideal than in the U_3_Si_2_ type^71^ (vide infra), leaving sizable residual voids. This is reflected in the lower Kitaigorodskii’s packing index (KPI)^72^ of the Er_3_Ni_2_-type Sm_3_Pt_2_ structure, 76.5%, compared to 78.6% of U_3_Si_2_-type Gd_3_Pd_2_.

Seven more structure types with 60 at. % rare earth have been reported in the literature across the lanthanide series with other transition metals and are briefly discussed here to clarify the structural tendencies at this stoichiometry. Among them, the Pd series is the one with the most representatives. In addition to the Er_3_Ni_2_ type, reported for Eu and Y, several lanthanides adopt the U_3_Si_2_ type, namely Gd, Dy, Ho and Er, whereas Tb_3_Pd_2_ adopts its own related type.^54^ It should, though, be noted that the difference between the Tb_3_Pd_2_ and U_3_Si_2_ types is very minor and is expressed mainly in a slight distortion of the characteristic building blocks (Figure 4). Both crystal packings consist exclusively of bicentered Pd_2_@Ln_12_ equatorially tetracapped tetragonal prisms (alternatively described as fused tricapped trigonal prisms), equivalent to those discussed above. Although these units are more regular than those observed in Ln_3_Pt_2–*x*_, their bases still exhibit some degree of corrugation.

**Figure 4:**
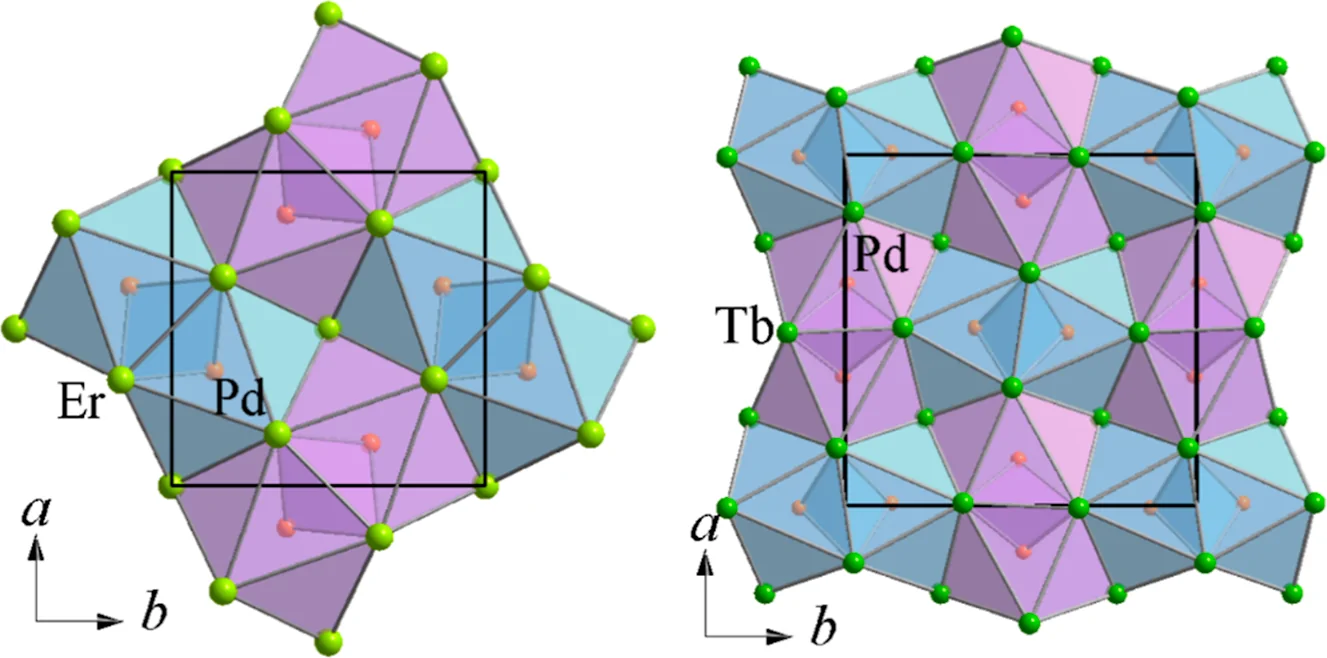
Polyhedral packing in the crystal structures of Er_3_Pd_2_ (U_3_Si_2_ type) and Tb_3_Pd_2_.^54^

**Y**_**3**_ **Rh**_**2**_ ^52^ represents the quintessence of structural complexity among the Ln_3_T_2_ types discussed above, combining their characteristic features. Similar to Ln_3_Pt_2–*x*_, the crystal structure of Y_3_Rh_2_ fits well into a bilayered architecture although with BBA sequence. Interestingly, it crystallizes in the same space group (SG) *I*4/*mcm*, but its unit cell is almost doubled along the *c* direction, although no symmetry relationships can be identified. One slab consists of square Rh@Y_8_ prisms (Figure 5, slab A) surrounded by regular Rh_2_-bicentered equatorially tetracapped tetragonal prisms Rh_2_@Y_12_. The second slab (Figure 5, slab B) is more complex containing identical tetracapped tetragonal prisms Rh_2_@Y_12_ surrounded by a complex bicentered polyhedron Rh_2_@Y_14_, which can be described as a fusion of tetragonal antiprism Rh@Y_8_ and bicapped distorted tetragonal prism Rh@Y_10_ (Figure S2). The latter have strictly parallel bases, a feature not observed in any of the preceding structures allowing formation of stacks along the *c* axis. Consequently, this structure type may be viewed as an intermediate or possible distortion pathway between the U_3_Si_2_- and Ln_3_Pt_2–*x*_-type structures.

**Figure 5:**
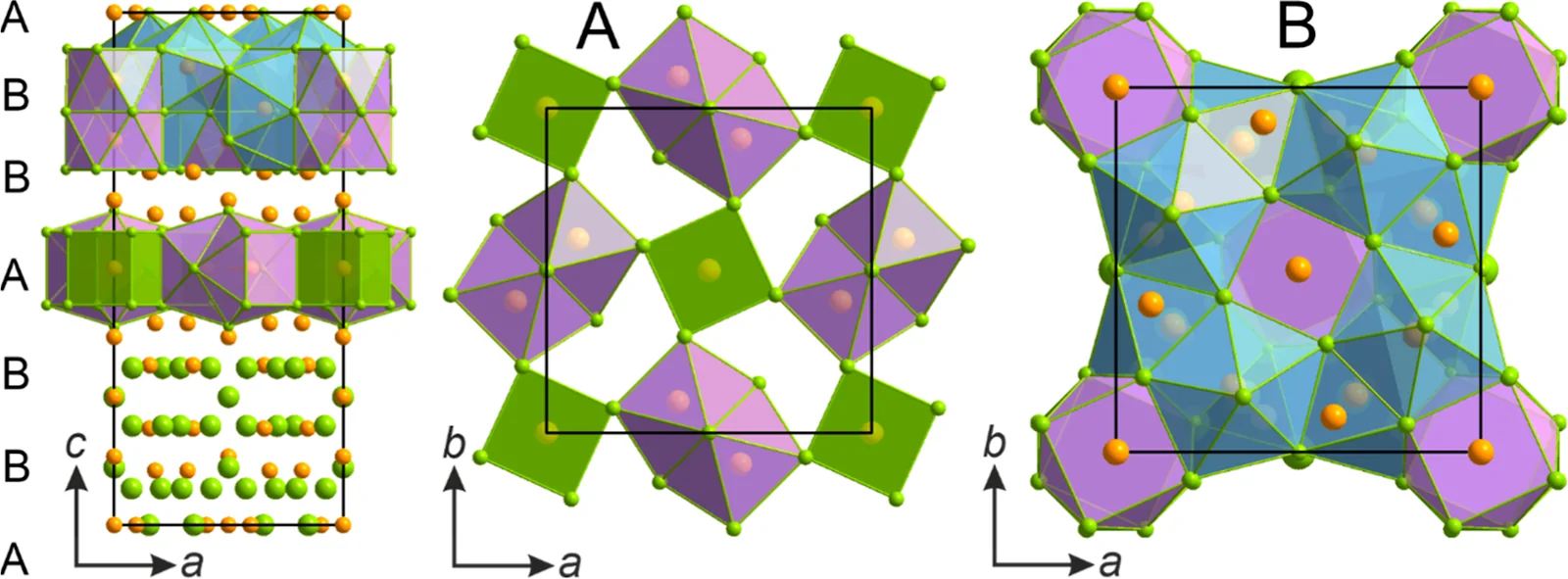
Atomic packing in the crystal structure of Y_3_Rh_2_ (left) and projections of the layers down the *c* axis. Ln atoms are shown green, and Pt—orange.

The next two types **Dy**_**3**_ **Ni**_**2**_ ^47^ and **Y**_**3**_ **Co**_**2**_ ^46^ stay somewhat apart and were introduced as CrB-related in the original report. Their characteristic feature is a tetrameric chairlike unit embedded in the rare-earth matrix (Figure 6a). These units stack through Ln-T and Ln–Ln connectivity, resulting in complex 3D arrangements. Coordination number of the Co and Ni in both structures is 9, but the coordination environment varies, depending on the position within the oligomeric chain, as those in the center have one more transition metal neighbor. The coordination polyhedra are always tricapped trigonal prisms, in which transition-metal atoms contribute to one another’s coordination environments. In other words, the tetrameric units can be described as four fused T@Ln_6_ trigonal prisms, highlighting both the main similarity and the defining feature of this group.

**Figure 6:**
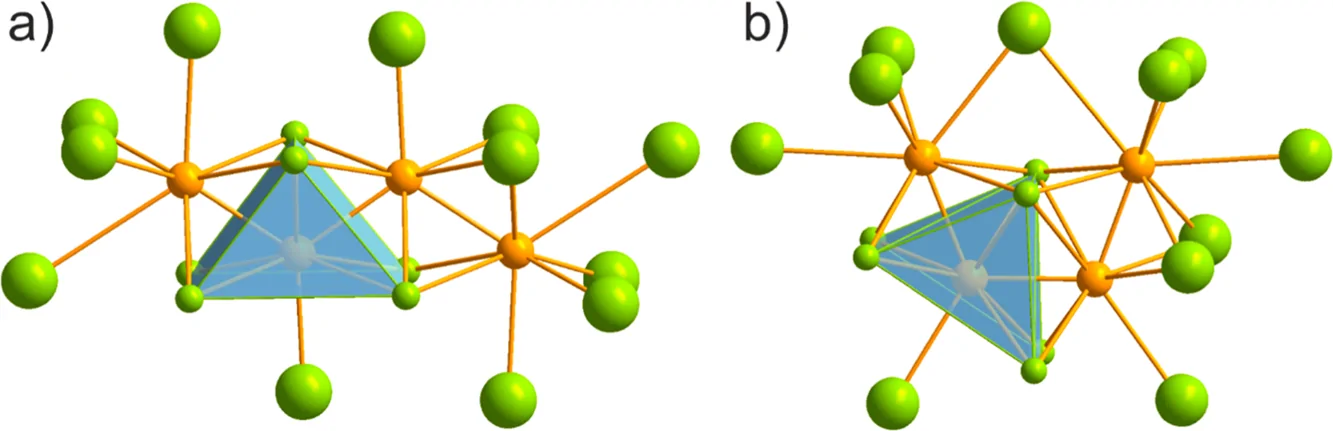
Dy_18_Ni_4_ unit in the crystal structure of Dy_3_Ni_2_ (a) and Y_17_Ni_4_ unit in the crystal structure of Y_3_Ni_2_ (b). Projections along the [0 1 0] and [−2 −7 7] directions, respectively.

Finally, the Y_3_Ni_2_ type^50^ represents their close relative, with the tetrameric unit adopting a boat conformation (Figure 6b). This geometry results in one capping Y atom being shared by the terminal Ni positions. The tetrameric Ni_4_ unit is highly asymmetric, with Ni–Ni distances ranging from 2.51 to 2.64 Å. Notably, Co–Co contacts in Y_3_Co_2_ are as short as 2.37 Å, pointing toward significant homoatomic interactions. For comparison, Ni–Ni contacts in Dy_3_Ni_2_ exceed 2.6 Å, despite the nearly identical Co and Ni covalent radii.^73^ Overall, these three types, from their local coordination environment to their packing motifs, indicate the existence of distinct structural subgroups within the Ln_3_T_2_ family. The possible occurrence of trimeric fused units and other variations within this family remains an open question.

The **Er**_**3**_ **Ru**_**2**_ type^74^ should also be mentioned briefly here. This structure type was reported for several rare-earth ruthenides and exhibits markedly different structural motifs, based on alternating rows of stacked trigonal prisms and antiprisms propagating along the *c* axis (Figure 7). However, closer inspection reveals unrealistically short Ru–Ru separations of approximately 2 Å, suggesting substantial underoccupation of one Ru site. A similar situation has been observed for La_2_Co_1.7_ ^75,76^ or Er_6_Co_5–*x*_,^54^ where apparently short transition-metal contacts are associated with partial occupancy and/or modulation. Therefore, the actual compositions of the reported Er_3_Ru_2_-type phases are likely slightly more Ln-rich than the ideal Ln_3_Ru_2_ formula. Further experimental work is required to clarify the crystal chemistry and homogeneity ranges of these phases.

**Figure 7:**
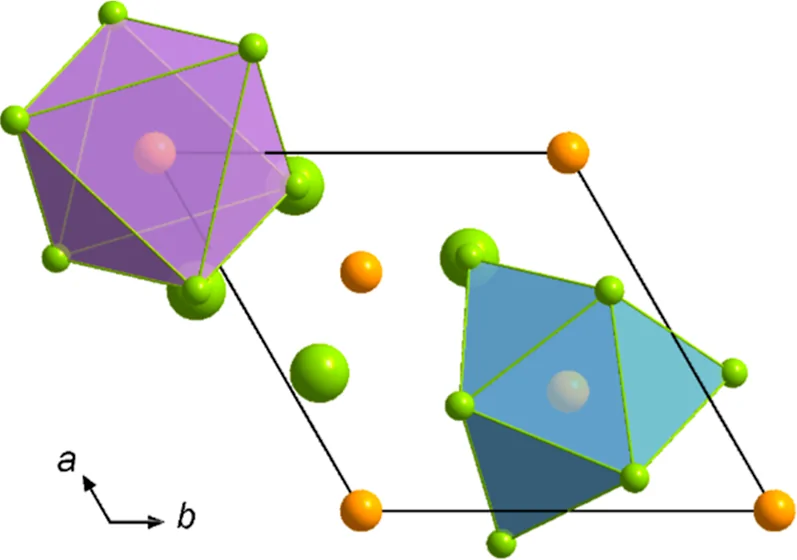
Atomic packing in the crystal structure of Er_3_Ru_2_. Tricapped trigonal prisms are marked blue and trigonal antiprisms—lavender.

### Ln_5_Pt_3_

The next series of compounds occurs only 2.5 at. % away from Ln_3_T_2_, at the Ln_5_T_3_ composition. In our samples, Ln_5_Pt_3_ phases were usually found in equilibrium with Ln_3_Pt_2_. La, Pr and Nd crystallize in the Pu_5_Rh_3_ structure type, similarly to Ce, whereas Sm unexpectedly adopts the Mn_5_Si_3_ type, showing an early change in the trend. It should also be noted that another closely related variant of Pu_5_Rh_3_–Cr_5_B_3_ has been observed for Yb_5_Au_3_^77^ and Yb_5_Ag_3_.^62^ Pu_5_Rh_3_(W_5_Si_3_) and Cr_5_B_3_ are homeotypic phases and have been analyzed in detail as high-temperature (*ht*) and low-temperature (*lt*) polymorphs of Ta_5_Si_3_, while Mn_5_Si_3_ phase was speculated to be a metastable/stabilized high-temperature variant.^78^

The crystal structure of **Nd**_**5**_ **Pt**_**3**_ can be conveniently described in terms of checkered chains composed of face-sharing Pt@Nd_8_ square antiprisms and edge-sharing Nd@Pt_4_ tetrahedra (Figure 8a), extending along the *c* axis. For presentation purposes, the former may be formally fragmented into face-sharing dimers of fused trigonal tricapped prisms Pt@Nd_8_Pt, similar to those observed in the preceding compounds. This fragmentation is purely formal, since all Pt–Pt distances in the chain are equal slightly exceeding 3.28 Å. The Pt positions not involved in these chains adopt the now familiar trigonal-prismatic coordination Pt@Nd_9_ although one Nd atom is located in the second coordination sphere. Otherwise this coordination polyhedron is closer to the usual square antiprism. The slightly higher proportion of Ln atoms in this compound is reflected in a weaker connectivity between the different Pt-centered units. In contrast to the fused oligomeric units observed above, Pt trigonal prisms share only common edges with identical neighboring units, highlighting a gradual transition toward the connectivity modes observed in Ln-richer phases.^24^

**Figure 8:**
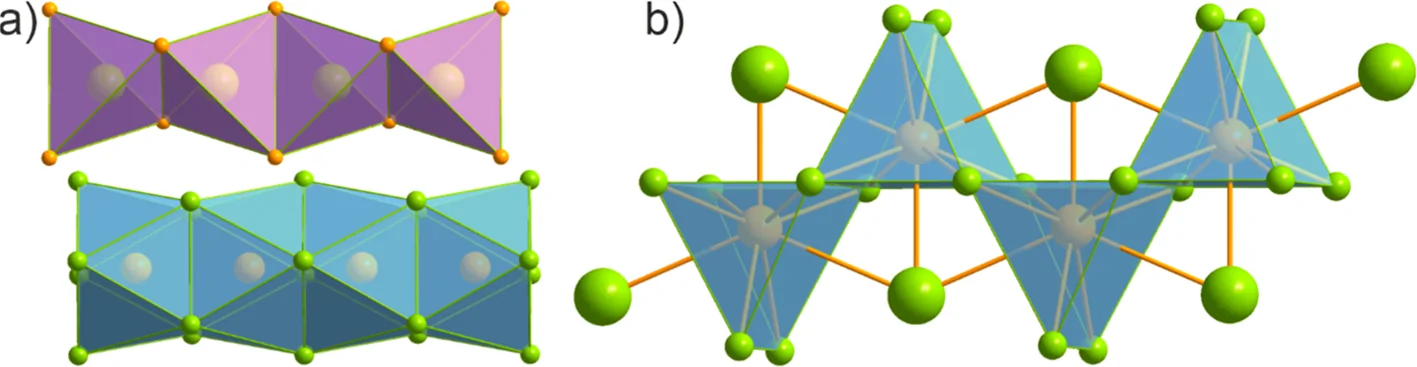
Polyhedral motifs in the crystal structures of (a) Nd_5_Pt_3_ and (b) Sm_5_Pt_3_. Ln atoms are green and Pt—orange. Projections along the [1 3 0] and [0 7 1] directions, respectively.

Interestingly, the Nd_5_Pt_3_ belongs to the same homologous series as Y_3_Rh_2_–R_5*n*+6_T_3*n*+5_.^52^ The Ln_5_Pt_3_ compounds crystallizing in the Pu_5_Rh_3_ structure type represent the limiting member with *n* = ∞, whereas Y_3_Rh_2_ corresponds to *n* = 3. The only other known representative with *n* = 5 (Pu_31_Pt_20_) was not observed with any rare earth element so far.

The crystal packing of **Sm**_**5**_ **Pt**_**3**_ (Figure 8b) is built exclusively from edge-sharing Pt-centered tricapped trigonal prisms forming chains along the *c* axis. These chains are connected to four equivalent chains through additional edge-sharing. No close Pt–Pt contacts can be observed, with the shortest distances approaching 3.6 Å. Alternatively, the structure may be described as a stacking of Sm@Pt_6_ trigonal antiprisms, each sharing edges with three equivalent units. In both representations, this packing leaves trigonal antiprismatic tunnels built of Sm atoms and extending along 00*z*. Considering these substantial changes in unit connectivity, it is unsurprising that, despite the same composition, no symmetry relationships can be identified between the two structures, following the trend observed for the Ln_3–*x*_Pt_2_/Ln_3_Pt_2_ polymorphs.

The last but not least interesting variant in this group is the crystal structure of **Yb**_**5**_ **Au**_**3**_ (Cr_5_B_3_ structure type). This crystal structure, similarly to Ln_3–*x*_Pt_2_, exhibits a bilayered architecture. One of the layers is composed of edge-sharing tetragonal antiprisms (Figure 9), while the other—of dimeric motifs identical to those in Figure 4. The second layer ultimately belongs to the U_3_Si_2_ type.^71^ The layers’ composition and connectivity represent a somewhat simplified version of Ln_3–*x*_Pt_2_, revealing deep connections within the Ln-T systems.

**Figure 9:**
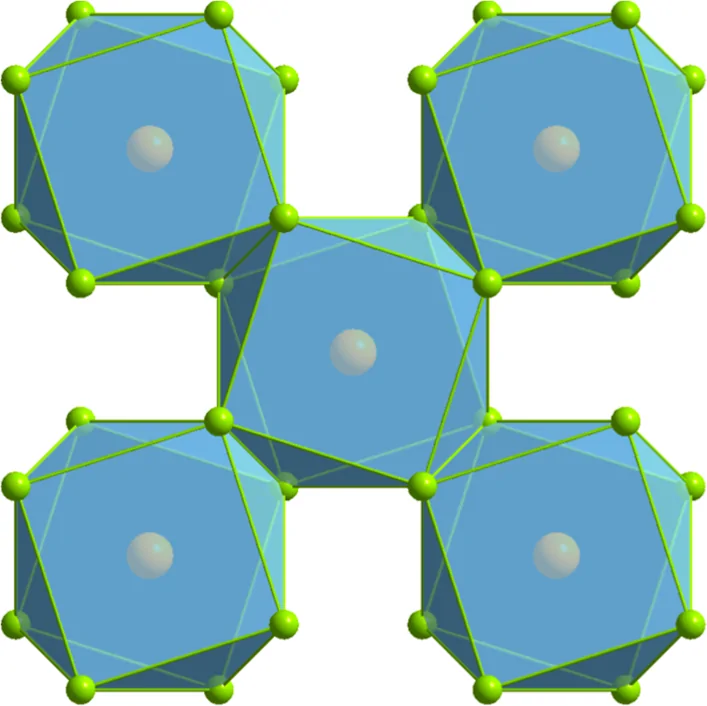
Tetragonal antiprismatic motif in the crystal structure of Yb_5_Au_3_. Projection along the *c* axis.

### Ln_2_Pt

The last compounds observed in this composition region are Co_2_Si-type **Ln**_**2**_ **Pt** phases, found only for Pr, Nd and Sm. The Co_2_Si type is closely related to the very common and highly representative type TiNiSi, which is known for its considerable flexibility toward compositional and geometric variations.^79^ In Ln_2_Pt, the Pt atoms occupy the position of more electronegative Si, whereas the Ln atoms occupy the Co sites.

In general, local coordination and connectivity in Sm_2_Pt closely resemble those in Sm_5_Pt_3_, as both contain edge-sharing trigonal-prismatic chains; however, in Sm_2_Pt, these chains are now corrugated (Figure 10). Owing to this corrugation, the connectivity between the prisms is somewhat weakened, as the number of shared capping atoms is reduced by 50%. In addition, the slightly higher Sm content affects the connectivity between the chains, which no longer share edges or faces but are linked via additional Sm–Pt contacts. The chains are not yet completely separated, however, since the Sm atoms coordinated in Pt@Sm_6_ prisms in one chain serve as capping atoms for a neighboring one. Consequently, all Pt–Pt contacts slightly exceed 4 Å, continuing the gradual increase observed in the sequence Nd_5_Pt_3_–Sm_5_Pt_3_–Sm_2_Pt.

**Figure 10:**
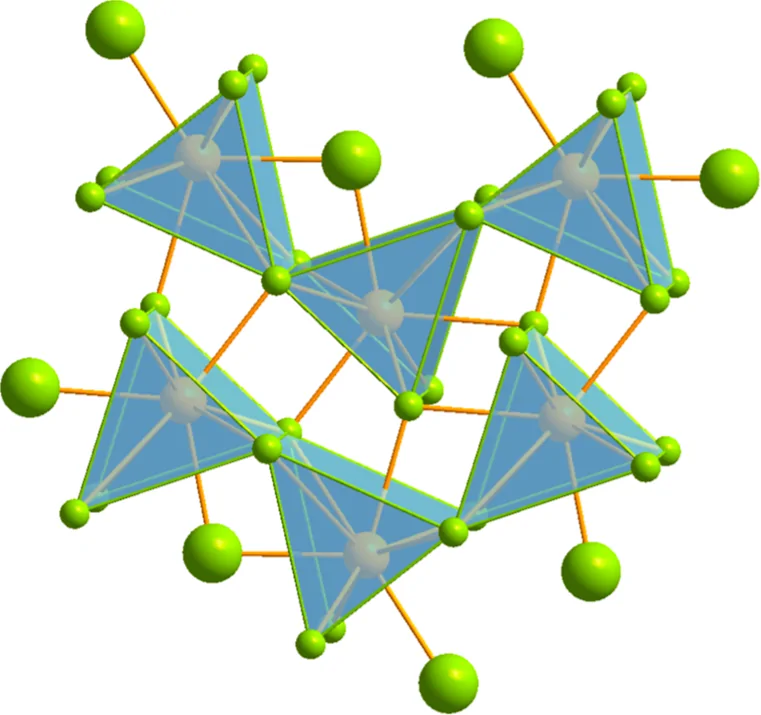
Trigonal prismatic motifs in the crystal structure of Sm_2_Pt. Projection along the *b* axis.

### Polymorphism in Ln_5_Pt_3_

Following the Ln_3_Pt_2_ series, the Ln_5_T_3_ compounds also exhibit structural variability, although they are restricted to only two structure types, tetragonal Pu_5_Rh_3_ and hexagonal Mn_5_Si_3_, and two transition metals—Pt and Ir. Interestingly, both structure types were reported as polymorphs for the corresponding Ln_5_Ir_3_ compounds (see Table 1).

In the Ln_5_Pt_3_ series, Ce_5_Pt_3_ was known to crystallize in the Pu_5_Rh_3_ type, whereas all heavy lanthanides and Y were reported in the Mn_5_Si_3_ type. However, no data were available for the remaining lanthanides, and the point of transition between these structure types remained unclear. Due to experimental difficulties in preparing Sm samples, DFT calculations were employed to examine the structural preference of Sm5Pt3, suggesting that the tetragonal form of the Pu5Rh3-type structure is the preferred one (Table 2). Surprisingly, a minor fraction of Sm_5_Pt_3_ detected in the as-cast samples was assigned to the hexagonal Mn_5_Si_3_ type. To test this trend experimentally, a corresponding Gd sample was prepared and annealed at 600 °C, confirming the existence of a tetragonal low-temperature (*lt*) polymorph in addition to the reported high-temperature (*ht*) hexagonal form.^59^ Although the heavier lanthanides were initially beyond the scope of this experimental work, they were also examined by DFT methods, which suggest that the Pu_5_Rh_3_ type is the thermodynamically preferred structure for all of them (Table 2).

**Table 2:** Total Energies and Optimized Volumes for the Series Ln_5_Pt_3_

	type	Nd	Sm	Gd	Tb	Dy	Ho	Er	Tm	Yb	Lu	Y
*E*/f.u. (eV)	Mn_5_Si_3_	–48.67	–48.72	–48.93	–48.95	–48.97	–48.99	–49.03	–49.04	–49.12	–49.13	–58.38
*V*/f.u. (Å^3^)		213.29	204.39	196.80	193.98	191.259	188.749	186.41	183.88	182.01	179.60	192.80
*E*/f.u. (eV)	Pu_5_Rh_3_	–48.97	–49.01	–49.20	–49.21	–49.21	–49.22	–49.24	–49.24	–49.32	–49.32	–58.63
*V*/f.u. (Å^3^)		217.09	209.19	202.10	199.04	196.69	194.36	191.80	189.87	187.76	185.26	198.20
Δ*E* (eV)		0.31	0.29	0.27	0.25	0.24	0.23	0.21	0.21	0.20	0.19	0.25
Δ*V* (Å^3^)		–3.80	–4.79	–5.30	–5.06	–5.43	–5.61	–5.39	–5.99	–5.75	–5.66	–5.40

As established above (Table 2), the *ht* form of Ln_5_Pt_3_ has a higher total energy but a smaller formula unit volume. Therefore, we examined the changes in the bonding patterns in more detail, using the polymorphs of Gd_5_Pt_3_ as an example. Unsurprisingly, owing to its high proportion, Gd participates in more than 95% of the bonding interactions in both structures, whereas Pt–Pt contacts are rather rare and weak (Table 3). The main difference is the higher contribution of Gd–Pt heteroatomic bonding in the *lt* polymorph, accompanied by a significant decrease in homoatomic Pt–Pt bonding.

**Table 3:** Distances, −ICOHP/Bond, Cumulative −ICOHP and Pair Contributions in Total Bonding

contact	distance (Å)	–ICOHP (eV/bond)	no./cell	–ICOHP (eV/cell)	%
*lt*-Gd_5_Pt_3_ (tetragonal)					
Pt–Pt	3.233	**0.38**	**4**	**1.52**	**1.33**
Gd–Pt	2.881	1.14	16	18.30	16.08
Gd–Pt	2.983	0.96	16	15.41	13.54
Gd–Pt	3.069	0.84	16	13.41	11.78
Gd–Pt	3.168	0.78	16	12.41	10.90
Gd–Pt	2.956	1.23	16	19.69	17.30
Gd–Pt	2.999	1.21	16	19.36	17.01
		**1.027**	**96**	**98.57**	**86.62**
Gd–Gd	3.233	0.27	4	1.10	0.97
Gd–Gd	3.477	0.15	16	2.42	2.13
Gd–Gd	3.536	0.25	16	3.98	3.50
Gd–Gd	3.588	0.09	8	0.70	0.62
Gd–Gd	3.753	0.16	16	2.64	2.32
Gd–Gd	3.761	0.18	16	2.87	2.52
		**0.180**	**76**	**13.71**	**12.05**
*ht*-Gd_5_Pt_3_ (hexagonal)					
Pt–Pt	3.559	**0.42**	**6**	**2.54**	**4.74**
Gd–Pt	2.959	0.82	12	9.82	18.34
Gd–Pt	3.079	0.96	6	5.78	10.80
Gd–Pt	3.435	0.36	12	4.37	8.16
Gd–Pt	3.026	1.00	24	24.05	44.93
		**0.815**	**54**	**44.01**	**82.22**
Gd–Gd	3.138	0.49	4	1.95	3.65
Gd–Gd	3.466	0.14	6	0.84	1.57
Gd–Gd	3.721	0.04	12	0.46	0.86
Gd–Gd	3.669	0.16	24	3.73	6.96
		**0.152**	**46**	**6.98**	**13.04**

In both structures, rather short Gd–Gd contacts are also observed: 3.233(1) Å in the tetragonal structure and 3.138 Å in the hexagonal one.^59^ In both cases, the corresponding—ICOHP values are comparable to those of the Pt–Pt contacts in the same structures and are significantly larger than those of the other Gd–Gd pairs. This behavior may be related to the stronger ionization of the Gd atoms, each surrounded by four Pt atoms in the tetragonal structure, together with the specific coordination of the short Gd–Gd contact, which is surrounded by only two Pt atoms. The situation in the hexagonal structure is even more pronounced: the Gd atoms involved in the short contact are each surrounded by six Pt atoms, while the Gd–Gd contact itself is coordinated by three Pt atoms. A similar bond-shortening effect has been observed in the polar intermetallic Na_8_Au_9.8_Ga_7.2_ and related structures, where it was associated with limited bond coordination.^80^

## Concluding Remarks

Each series displays a unique structural footprint among the Ln-T systems, including compositions closer to 70% Ln discussed previously.^24^ This highlights the strong sensitivity of these compounds to geometric factors.

The Er_3_Ni_2_ structure type is adopted by all platinides with light lanthanides from La to Gd, being the only structure type that remains common across the entire series of lighter lanthanides up to Gd for any transition metal. Within this series, Sm_3_Pt_2_ was the only compound that had not yet been structurally characterized, despite its existence being predictable from systematic trends across the Ln–Pt systems. The component distribution in this structure type is notable: larger light rare-earth cations allow a broader range of transition metals, including Au, Pd, Pt, Rh, Ir, and Ni, whereas heavy-lanthanide representatives are currently known mainly with Ni or Co/Ni mixtures.^81^ The latter may be regarded as pseudobinary phases with no ordering of a third component.

The current structural overview suggests that the Er_3_Ni_2_ structure type remains significantly underexplored. Known examples include Rh-containing phases with light lanthanides, Ni-containing phases with heavy lanthanides and Yttrium, and isolated representatives with Ir, Pd, and Au. It appears reasonable that light lanthanides prefer this type with the heavier transition metals, while heavy lanthanides favor Ni-containing analogs, pointing to a clear geometric correlation. However, Y_3_Pd_2_ is an important outlier, crystallizing in the Er_3_Ni_2_ type together with Eu_3_Pd_2_. Moreover, multiple other types have been reported for the same composition, e.g., U_3_Si_2_, Y_3_Rh_2_, Tb_3_Pd_2_, etc. The latter was accessed only at lower temperatures via soft chemistry methods,^54^ suggesting that the entire area may require further experimental clarification.

In contrast to Er_3_Ni_2_-type phases, the higher-temperature polymorph—the Pr_3_Pt_2–*x*_ type currently appears to be characteristic of Pt systems only. Its existence was confirmed for Ce, Pr, Nd, and Sm, but no traces were observed for La or Eu. Although there is no obvious geometric or electronic reason preventing full stoichiometry, the Pr_3_Pt_2–*x*_ structure type appears to tolerate a finite compositional range and may approach the ideal Ln_3_Pt_2_. The electronic structure calculations do not reveal a pronounced pseudogap or another distinct feature favoring a specific Pt-deficient composition. As this phase is the high-temperature polymorph, partial Pt occupancy may additionally be favored by configurational entropy as e.g., in Dy_2+*x*_Pd_1–*x*_ phases.^24^ The observed nonstoichiometry is therefore better regarded as compositional flexibility of the structure than as a strict packing or electron-count requirement.

The Ln_3_Pt_2–*x*_ phases show that structural motifs commonly associated with main-group chemistry can also be accessed using transition metals only. This is particularly important for expansion into ternary regions. While *d*-*d*-*p* intermetallics are well represented, particularly TiNiSi structure type and its close relatives,^79,82^ *d*(*f*)-*d*-*d* ternary compounds are rarer and usually represent disordered pseudobinary solid solutions, e.g., in the common MgCu_2_, CaCu_5_ or Cu_3_Au structure types.^83–85^ From the distinct ternary phases, we can highlight Pr_6_Fe_13_Ge, MgCu_4_Sn, or Gd_4_RhIn structure types where the *p* element could fully be substituted by a late transition metal as a rule from groups 10 or 11.^86–88^

The Pu_5_Rh_3_ structure type was relatively rare among lanthanides and, until recently, was mainly associated with Ir systems, where it forms across nearly the entire lanthanide series except Eu (Table 1). Previously, Ce_3_Pt_2_ ^48^ was the only non-Ir exception. In the present work, related platinides were also identified for La, Pr and Nd. Starting from Sm, however, the series switches to the Mn_5_Si_3_ structure type, which then extends to Lu. A similar transition is known in Ln-Ir systems beginning at Tb, where Mn_5_Si_3_-type phases coexist with Pu_5_Rh_3_-type phases as high-temperature polymorphs. In Pt systems, the transition occurs earlier, already at Sm, and it has yet to be proved experimentally if no *ht* phase exists at least for Gd_5_Ir_3_.

The polymorphism observed for these platinides is not unusual among binary Ln-T systems. Ln-Pd systems, for example, display pronounced polymorphism in which phases share identical *ht* forms but differ in their *lt* structures. Some low-temperature polymorphs can only be accessed via soft-chemistry routes, such as Er_17_Pd_7_ (*lt* form of Er_2.11_Pd_0.89_) or Tb_7_Pd_3_ (*lt* form of Tb_2+*x*_Pd_1–*x*_).^24^ Interestingly, the Er_17_Pd_7_-type structure has also been reported as the *lt* form of Pr_5_Co_2_ despite the significantly different Ln/T size ratio.

The Co_2_Si structure type was identified for Pr_2_Pt, Nd_2_Pt and Sm_2_Pt, extending the known series beyond the heavy lanthanide platinides. This structure type was also characteristic of Ln-Au systems across nearly the entire series, except for two divalent lanthanides. The structural fingerprint of Ln_2_Pt with the light lanthanides resembles that of Mn_5_C_2_-type compounds known with Co,^24^ although those have not yet been reported for heavy lanthanides. Notably, Eu_2_Pt has never been observed, whereas Yb_2_Pt was assigned to the anti-PbCl_2_ structure type in line with Yb_2_Au and Y_2_Au, which is essentially the same as Co_2_Si.

Overall, the present results show that binary Ln-T systems remain far from fully characterized. Despite the substantial amount of new data, many gaps remain. Some missing compounds and structural trends can be predicted directly from the summarized structural tables, whereas others are less obvious and require experimental verification. Particularly complex are the heavy-lanthanide Ln_3_T_2_ systems, which show extensive structural diversity, with Y frequently deviating from expected trends. In several cases, further work will also be required to distinguish true absence of a phase from synthetic limitations, polymorphism, nonstoichiometry, or metastability. Although ternary systems have been widely studied and many structural trends have been established, the corresponding binary systems still contain major gaps in fundamental knowledge. Closing these gaps is therefore essential, since binary phases provide the structural and chemical reference points needed to understand more complex ternary and multicomponent intermetallics.

## Supplementary Material



## Notes

Additional crystallographic information, Unit cell refinement results, DFT optimized unit cell data and EDX results (PDF)
