# Supplementary material for: Mapping the Hidden Landscape of Ln-Rich Platinides
Source: Inorg Chem. 2026 Aug 24;65(43):25223–33. doi: 10.1021/acs.inorgchem.6c03130 (PMC13733265; doi:10.1021/acs.inorgchem.6c03130)
Supplement: Supplementary file 1 [file ic6c03130_si_001.pdf]

Mapping the Hidden Landscape of Ln-Rich Platinides

Volodymyr Smetana,[1] Vitalii Shtender,[2] Gerd H. Meyer,[1] Anja-Verena Mudring[1,3*]

[1]*Department of Biological and Chemical Engineering and iNANO, Aarhus University, 8000 Aarhus C, Denmark.*

[2]*Department of Chemistry – Ångström Laboratory, Uppsala University, Box 538, SE-751 21 Uppsala, Sweden.*

[3]*Department of Physics, Umeå University, 90187 Umeå, Sweden. Email: anja-verena.mudring@bce.au.dk*; mailto:*anja-verena.mudring@umu.se*

Supporting Information

Table of content

1. Crystallographic details and refinement parameters P S2
2. DFT-optimized unit cell parameters for Ln_5Pt_3 P S4
3. Crystallographic data for selected binary Ln-Pt compounds P S5
4. EDX results P S6
5. Supporting structural images P S6

Table S1. Crystallographic Details and Refinement Parameters for Ce_3Pt_{2-x}, La_5Pt_3, Nd_3Pt_{2-x}, Pr_5Pt_3, Sm_2Pt and Sm_3Pt_2 (SCXRD data).

	Ce_3Pt_{2-x}	La_5Pt_3	Nd_3Pt_{2-x}	Pr_5Pt_3	Sm_2Pt	Sm_3Pt_2	Gd_3Pt_2
**Empirical formula**	$Ce_3Pt_{1.98}$	La_5Pt_3	$Nd_3Pt_{1.98}$	Pr_5Pt_3	Sm_2Pt	Sm_3Pt_2	Gd_3Pt_2
**Formula weight**	806.37	1279.82	819.30	1289.82	495.79	841.23	861.93
**Temperature**	298(2)	298(2)	298(2)	298(2)	298(2)	298(2)	293(2)
**Wavelength**	0.71073	0.71073	0.71073	0.71073	0.71073	0.71073	1.54184
**Crystal system**	Tetragonal	Tetragonal	Tetragonal	Tetragonal	Orthorhombic	Trigonal	Trigonal
**Space group**	I4/mcm	P4/ncc	I4/mcm	P4/ncc	Pnma	R-3	R-3
**a, Å**	12.032(4)	11.524(1)	11.8673(9)	11.337(3)	7.218(2)	8.804(2)	8.7388(4)
**b, Å**	12.032(4)	11.524(1)	11.8673(9)	11.337(3)	4.828(2)	8.804(2)	8.7388(4)
**c, Å**	14.52(1)	6.785(1)	14.439(2)	6.586(3)	8.919(3)	16.833(6)	16.584(1)
**Volume, Å^3**	2103(2)	900.9(3)	2033.4(4)	846.4(6)	310.8(2)	1129.8(7)	1096.8(1)
**Z**	16	4	16	4	4	9	9
**Density (calculated), g/cm3**	10.189	9.436	10.705	10.121	10.596	11.127	11.744
**μ, mm^{-1}**	77.735	69.439	84.237	77.454	81.853	89.854	362.524
**F(000)**	5253	2076	5353	2116	808	3078	3132
**θ range,**	2.394º to 25.984º	2.500º to 27.497º	2.427º to 25.987º	2.541º to 27.490º	3.631º to 27.436º	2.933º to 25.989º	6.429º to 78.946º
**Index ranges**	-14<h<13 -14<k<14 -17<l<17	-14<h<12 -14<k<14 -7<l <8	-14<h<14 -14<k<14 -17<l<13	-14<h<8 -11<k<14 -8<l<8	-7<h<9 -6<k<6 -11<l<11	-10<h<9 -9<k<9 -20<l<16	-11<h<10 -11<k<11 -18<l<20
**Reflections collected**	3159	1931	3301	2643	1194	1382	5035
**Independent reflections**	578	522	567	492	397	468	533
**Observed reflections**	346	354	454	344	344	433	502
**Refinement method**	Full-matrix least squares on F²						
**Data/restraints/parameters**	578/0/33	522/0/20	567/0/33	492/0/20	397/0/19	468/0/24	533/0/24
**Goodness-of-fit on F^2**	1.017	1.016	0.965	1.574	1.129	1.175	1.112
**Final R indices [I>2σ(I)]**	R1=0.0595 wR2=0.1212	R1=0.0381 wR2=0.0775	R1=0.0315 wR2=0.0750	R1=0.0399 wR2=0.1305	R1=0.0335, wR2=0.0810	R1=0.0525, wR2=0.1384	R1=0.0333, wR2=0.0711
**R indices (all data)**	R1=0.1299 wR2=0.1359	R1=0.0755 wR2=0.0849	R1=0.0438 wR2=0.0781	R1=0.0791 wR2=0.1369	R1=0.0425 wR2=0.0831	R1=0.0573 wR2=0.1416	R1=0.0363 wR2=0.0725
**R_{int}**	0.1934	0.0598	0.0463	0.0672	0.0294	0.0430	0.0946
**Largest diff. peak and hole, e$^-$/Å^3**	4.68 and -3.68	3.60 and -2.34	2.69 and -4.27	3.03 and -2.81	2.57 and -2.66	3.10 and -4.28	2.16 and -2.58

Table S2. Crystallographic Details and Refinement Parameters for Gd_5Pt_3, Nd_2Pt, Nd_5Pt_3, Pr_2Pt, Sm_3Pt_{2-x} and Sm_5Pt_3 (PXRD data).

	**Gd_5Pt_3_*lt***	**Nd_2Pt**	**Nd_5Pt_3**	**Pr_2Pt**	**Sm_3Pt_{2-x}**	**Sm_5Pt_3**
**Empirical formula**	Gd_5Pt_3	Nd_2Pt	Nd_5Pt_3	Pr_2Pt	$Sm_3Pt_{1.92}$	Sm_5Pt_3
**Formula weight**	1371.484	483.558	1306.434	476.90	824.42	1337.03
**Temperature**	298(2)	298(2)	298(2)	298(2)	298(2)	298(2)
**Wavelength**	0.55942	0.55942	0.55942	0.55942	0.55942	0.55942
**Crystal system**	Tetragonal	Orthorhombic	Tetragonal	Orthorhombic	Tetragonal	Hexagonal
**Space group**	P4/ncc	Pnma	P4/ncc	Pnma	I4/mcm	P63/mcm
**a, Å**	11.092(1)	7.2859(8)	11.2689(8)	7.316(1)	11.646(2)	8.4886(6)
**b, Å**	11.092(1)	4.8606(5)	11.2689(8)	4.8766(7)	11.646(2)	8.4886(6)
**c, Å**	6.4662(8)	9.0014(9)	6.5850(6)	9.037(2)	14.595(5)	6.3237(6)
**Volume, $Å^3$**	795.6(2)	318.78(6)	836.2(1)	322.40(9)	1979.7(9)	394.62(6)
**Z**	4	4	4	4	16	2
**Density (calculated), g/cm^3**	11.450	10.076(2)	10.377	9.825(3)	11.064(6)	11.252(2)
**μ, mm-1**	49.008(12)	39.118(7)	41.817(7)	39.082	46.31(2)	46.578(8)
**F(000)**	2216	792	2136	784	5364	1088
**θ range**	1.5025º to 18.9925º	1.5025º to 18.9925º	1.5025º to 18.9925º	1.5025º to 18.9925º	1.5025º to 24.9925º	1.5025º to 18.9925º
**Generated hkl index ranges**	$0\leq h\leq 13$<br>$0\leq k\leq 9$<br>$0\leq l\leq 7$	$0\leq h\leq 8$<br>$0\leq k\leq 5$<br>$0\leq l\leq 10$	$0\leq h\leq 13$<br>$0\leq k\leq 9$<br>$0\leq l\leq 7$	$0\leq h\leq 8$<br>$0\leq k\leq 5$<br>$0\leq l\leq 10$	$0\leq h\leq 17$<br>$0\leq k\leq 12$<br>$0\leq l\leq 22$	$0\leq h\leq 10$<br>$-5\leq k\leq 0$<br>$0\leq l\leq 7$
**Generated hkl entries**	341	310	362	310	1014	142
**Refinement method**	Rietveld					
**Goodness-of-fit**	3.50350	1.94029	1.94029	1.46392	2.81381	3.36803
**Profile R indices**	R_p=0.10066<br>R_{wp}=0.13562	R_p=0.04041<br>R_{wp}=0.05585	R_p=0.04041<br>R_{wp}=0.05585	R_p=0.02964<br>R_{wp}=0.03801	R_p=0.06498<br>R_{wp}=0.08246	R_p=0.06604<br>R_{wp}=0.09238
**R_{Bragg}**	0.06227	0.02295	0.01840	0.01432	0.02120	0.03517

Table S3. DFT-optimized unit cell parameters for Ln_5Pt_3 in Mn_5Si_3 and Pu_5Rh_3 structure types.

Structure type	Parameter	Nd	Sm	Gd	Tb	Dy	Ho	Er	Tm	Yb	Lu	Y
Mn_5Si_3	*a* (Å)	8.6403	8.5871	8.5153	8.4873	8.4571	8.4268	8.3927	8.3575	8.3321	8.2991	8.4822
	c (Å)	6.5979	6.4014	6.268	6.2188	6.1755	6.1385	6.1118	6.0795	6.0546	6.0222	6.1886
	V (Å^3)	426.57	408.79	393.6	387.95	382.52	377.5	372.82	367.75	364.02	359.21	385.6
Pu_5Rh_3	*a* (Å)	11.411	11.273	11.143	11.084	11.037	10.988	10.945	10.895	10.851	10.804	11.064
	c (Å)	6.6688	6.5839	6.5105	6.4799	6.4582	6.4392	6.4042	6.3978	6.3785	6.3485	6.476
	V (Å^3)	868.35	836.72	808.4	796.14	786.75	777.43	767.21	759.46	751.03	741.04	792.81

Table S4. Crystallographic data for the binary Ln-Pt compounds around 60–70 at.% *Ln* observed in our work and corresponding literature data.

Composition	ST	SG	*a*, Å	*b*, Å	*c*, Å	Ref.
La_3Pt_2	Er_3Ni_2	$R\bar{3}$	9.096		17.303	48
La_3Pt_2	Er_3Ni_2	$R\bar{3}$	9.112(2)		17.367(7)	this work
La_7Pt_3	Th_7Fe_3	$P6_3mc$	10.336		6.516	89
La_7Pt_3	Th_7Fe_3	$P6_3mc$	10.371(2)		6.519(2)	this work
La_5Pt_3	Pu_5Rh_3	$P4/ncc$	11.524(1)		6.785(1)	this work
Ce_3Pt_2	Er_3Ni_2	$R\bar{3}$	8.981		17.078	48
$Ce_3Pt_{1.98}$	Pr_3Pt_{2-x}	$I4/mcm$	12.032(4)		14.52(1)	this work
Ce_7Pt_3	Th_7Fe_3	$P6_3mc$	10.204		6.399	89
Ce_7Pt_3	Th_7Fe_3	$P6_3mc$	10.205(5)		6.420(4)	this work
Ce_5Pt_3	Pu_5Rh_3	$P4/ncc$	11.405		6.648	90
Pr_3Pt_2	Er_3Ni_2	$R\bar{3}$	8.959		16.980	48
$Pr_3Pt_{1.99}$	Pr_3Pt_{2-x}	$I4/mcm$	11.931		14.447	22
$Pr_3Pt_{1.97}$	Pr_3Pt_{2-x}	$I4/mcm$	11.9444		14.488	22
Pr_7Pt_3	Th_7Fe_3	$P6_3mc$	10.137		6.376	89
Pr_7Pt_3	Th_7Fe_3	$P6_3mc$	10.164(3)		6.393(2)	this work
Pr_5Pt_3	Pu_5Rh_3	$P4/ncc$	11.337(3)		6.586(3)	this work
Pr_2Pt	Co_2Si	$Pnma$	7.316(1)	4.8766(7)	9.037(2)	this work
Nd_3Pt_2	Er_3Ni_2	$R\bar{3}$	8.893		16.871	48
$Nd_3Pt_{1.98}$	Pr_3Pt_{2-x}	$I4/mcm$	11.8673(9)		14.439(2)	this work
Nd_5Pt_3	Pu_5Rh_3	$P4/ncc$	11.2689(8)		6.5850(6)	this work
Nd_2Pt	Co_2Si	$Pnma$	7.2859(8)	4.8606(5)	9.0014(9)	this work
Nd_7Pt_3	Th_7Fe_3	$P6_3mc$	10.099		6.351	89
Sm_3Pt_2	Er_3Ni_2	$R\bar{3}$	8.804(2)		16.833(6)	this work
$Sm_3Pt_{1.97}$	Pr_3Pt_{2-x}	$I4/mcm$	11.646(2)		14.595(5)	this work
Sm_7Pt_3	Th_7Fe_3	$P6_3mc$	10.017		6.298	59
Sm_2Pt	Co_2Si	$Pnma$	7.218(2)	4.828(2)	8.919(3)	this work
Sm_5Pt_3	Mn_5Si_3	$P6_3/mcm$	8.489(3)		6.400(2)	this work
Sm_5Pt_3[#]	Pu_5Rh_3	$P4/ncc$	11.2776		6.5617	this work
Gd_2Pt	Co_2Si	$Pnma$	7.186	4.813	8.854	59
Gd_5Pt_3	Mn_5Si_3	$P6_3/mcm$	8.479		6.275	59
Gd_5Pt_3	Pu_5Rh_3	$P4/ncc$	11.092(1)		6.4662(8)	this work

[#] based on DFT

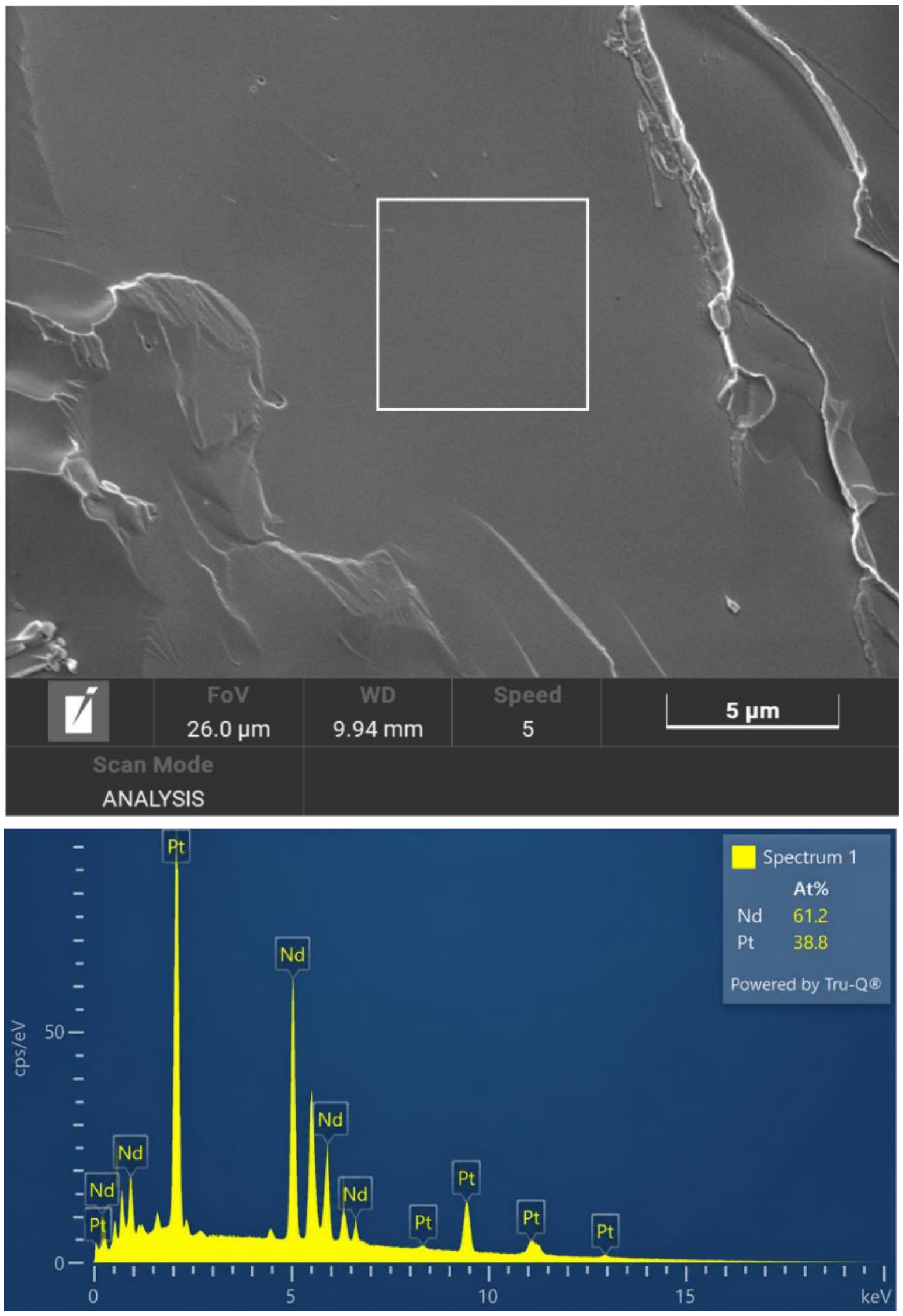

Figure S1. Backscattered electron image and an EDS spectrum acquired from a single crystal of $Nd_3Pt_{1.98}$.

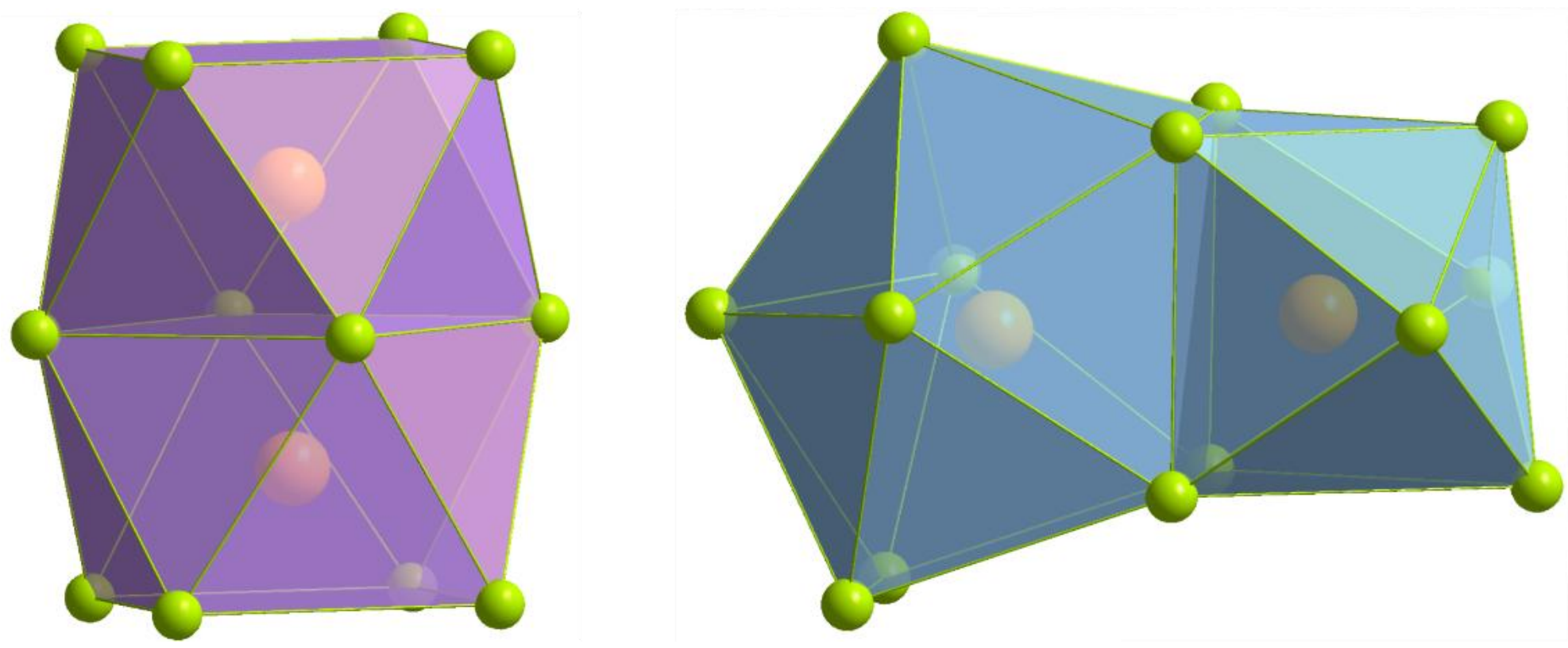

Figure S2. Coordination polyhedra Rh_2Y_{12} and $Rh_2@Y_{14}$ in the crystal structure of Y_3Rh_2.
